# Tenosynovial giant cell tumors as accidental findings after episodes of distortion of the ankle: two case reports

**DOI:** 10.1186/1752-1947-3-9331

**Published:** 2009-12-15

**Authors:** Christian Illian, Horst-Rainer Kortmann, Hans Otto Künstler, Ludger W Poll, Markus Schofer

**Affiliations:** 1 Berufsgenossenschaftliche Unfallklinik Duisburg GmbH, Grossenbaumer Allee 250, 47249 Duisburg, Germany; 2 Institut für Pathologie, Evangelisches Krankenhaus Bethesda, Duisburg, Heerstr. 219 47053 Duisburg, Germany; 3 Universitätsklinikum Marburg, Baldingerstrasse, 35043 Marburg, Germany

## Abstract

**Introduction:**

Tenosynovial giant cell tumors are benign tumors of uncertain pathogenesis. They occur in the joints, tendons and synovial bursas. Due to a high recurrence rate of up to 50%, some authors call a giant cell tumor a semimalignant tumor. To date, less than 10 cases of tenosynovial giant cell tumor of the ankle have been published in the international medical literature.

**Case presentation:**

In this case report, we present two patients with localized tumors that were detected accidentally after the occurrence of ankle sprains with persisting pain in the joint. The tumors were resected by open marginal surgery and regular follow-up examinations were carried out.

**Conclusions:**

We present an unusual occurrence of a tumor along with a possible follow-up strategy, which has not been previously discussed in the international literature.

## Introduction

A tenosynovial giant cell tumor (TGCT) is a benign tumor of uncertain pathogenesis. It occurs in the joints, tendons and synovial bursas. First described in the international literature by Jaffe *et al*. [1] in 1941, it has been given different names including nodular tenosynovitis or (pigmented) villonodular synovitis or tenosynovitis, and bursitis [1-5]. TGCT may be either localized or diffused. The localized type of the tumor is most commonly found in finger joints while subtypes of diffuse-type TGCT may be distinguished as intra-articular and extra-articular. The lesion may appear anywhere in the synovium, but in 80% to 90% of cases, it occurs in the hand joints, and infrequently in the knee and foot joints [6]. Due to the high recurrence rate of up to 50%, a correct classification of the tumor is essential. As a result of this, and also of the possible malignant degeneration of the tumor, some call the TGCT a semimalignant tumor [5-8],.

There has been no indication so far that specific age groups or gender have a higher incidence rate of acquiring the tumor. Studies described by Somerhausen *et al*. show 28 cases of the tumor occurring among women and 22 such cases occurring in men, which clearly shows no significant difference in incidence (binomial test, p = 0.479). Furthermore, some authors assume that the lesions are caused by an unknown agent while others consider them to be neoplastic [9,10,2].

Histologically, the growth of fibroblastic cells is followed by a reactive proliferation of histiocytic cells in the reticuloendothelial system. After phagocytosis of erythrocytes the cells undergo a transformation into hemosiderin-laden macrophages that merge into giant cells [10].

To date, less than 10 cases of TGCT of the ankle have been published in the international medical literature [4-7,9-13].

## Case presentation

### Case report 1

**Figure 1:**
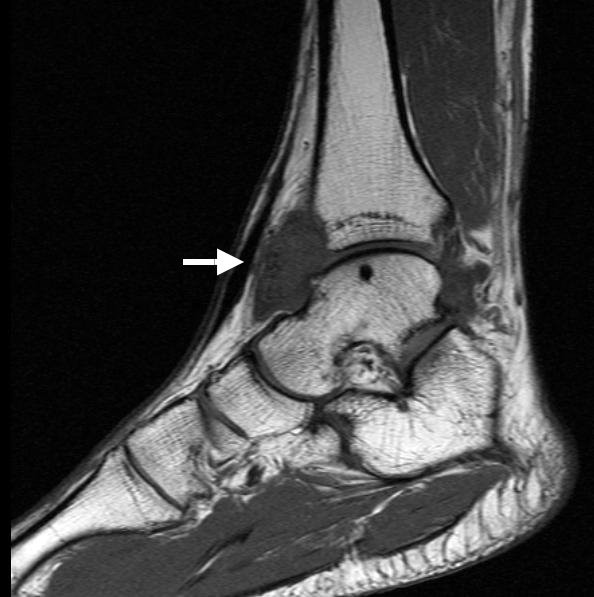
**Sagittal T1-weighted spin-echo MRI of the right ankle showing a well delineated giant cell tumor anterior to the ankle (arrow)**.

A 30-year-old Caucasian man suffered from a distortion of his right ankle seven months prior to presentation. The incident happened when he was at work. Due to persisting pain in his joint he first saw a general practitioner. An X-ray image of the patient's right ankle showed no pathological findings. The joint was immobilized for six weeks in a plaster cast, which was then followed by physical therapy. Six months after the therapy, however, the patient still suffered pain in his ankle with no sign of any improvement. A magnetic resonance imaging (MRI) scan revealed an unknown but well-circumscribed localized tumor at the ventral part of the ankle, coupled with focal bulging and erosion of the tibia and talus (Figure 1). The MRI detected no damage to the fibular collateral ligaments. On examination, about thirteen months after trauma of the ankle, tenderness to pressure was found at the ventral aspect of the right ankle next to the medial malleolus. A dorsal extension of the ankle was very painful. The collateral ligaments showed no insufficiency and a new X-ray still did not show any conspicuous findings. An ultrasound investigation showed a solid, homogeneous hypoechoic mass measuring 3.5 × 2.5 × 2 cm. It was not clear whether the tumor was directly connected to the joint. An impingement syndrome of the right ankle caused by a synovial hypertrophy was diagnosed preoperatively.

**Figure 2:**
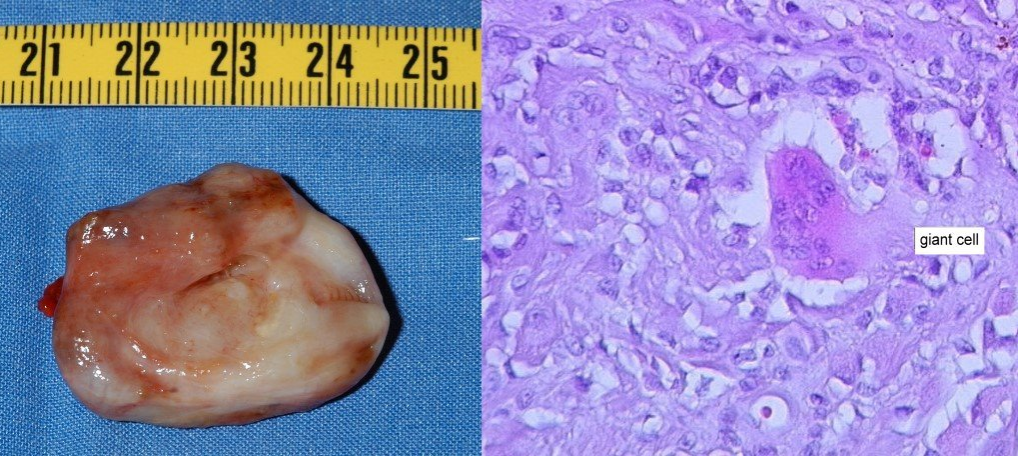
**Macroscopic and histological images of the TGCT**. **(A) **A macroscopic image of the tumor after resection. (B) Histological findings using hematoxylin and eosin staining of a giant cell.

The tumor was resected through a ventral access. A brownish yellow tumor that was mainly solid was found during surgery. The tumor showed adhesions to the capsular of the patient's ankle and the complete tumor was treated with marginal resection (Figure 2A). A small hypertrophy of the cartilage below the tumor was also removed. However, the complete cartilage of the joint was not damaged.

Microscopically, the tumor was partially encapsulated and composed of round to polygonal cells. Some were spindle cells and some were multinucleated giant cells (Figure 2B). The diagnosis of localized tenosynovial giant cell tumor of the tendon sheath was confirmed on histopathology. Results of special stains indicated the presence of iron in both mononuclear and multinucleated giant cells in cytologic and histologic preparations.

**Figure 3:**
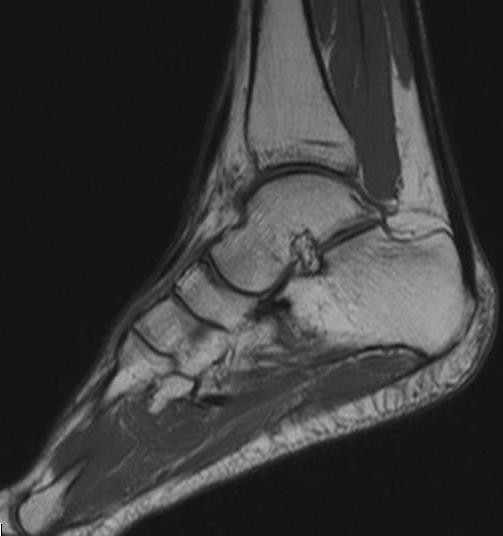
**An image of the follow-up MRI of the right ankle 24 months after surgery**. A sagittal T1-weighted spin-echo MR image showing subcutaneous scars anterior to the ankle.

During follow-up the patient presented no complications. Investigations three, six, 12, and 24, as well as the MRI scan conducted 24 months after surgery, showed no recurrence of the TGCT (Figure 3). To this day the patient is free of any symptoms.

### Case report 2

A 29-year-old Caucasian woman originally suffered from a distortion of her upper left ankle more than six years prior to presentation. The patient developed a chronic instability with recurrent distortions following the conservative treatment she underwent. After the most recent distortion, a computed tomography (CT) scan revealed substantial cystic lesions in the trochlea of her talus, synovial reaction with foreign tissue in the neighboring capsule and signs of a villonodular inflammation of the synovial membrane. On physical examination upon admission, however, the patient's gait pattern and mobility showed no abnormalities.

A lateral instability caused by ligament insufficiency was consequently found. Lateral stress views showed a 15-degree clear space widening and a talar shift of more than 10 mm.

The patient underwent an arthroscopy of the left ankle. The tumor was resected via open surgery. The articular surface of the talus and the distal tibia showed an extensive four-degree defect of the cartilage. Arthrotomy showed a brownish yellow tumor that was mainly solid attached to the ventral synovial tissue. This was entirely removed through a marginal resection. Additionally, these defects were smoothened and microfractured. The ligamental structures were not rebuilt because of advanced arthrosis of the patient's upper ankle. No complications occurred after the operation and the histological analysis identified the tumor as a localized TGCT.

Follow-up examinations after three, six, 12 and 24 months showed no indication of a recurrence of the tumor. An MRI scan 24 months after the operation showed no new tumor growth. However, a recurring pain in the patient's upper left ankle made another arthroscopy necessary. This procedure showed that a fibrocartilage had formed but no hypertrophic synovia was found to be present.

## Discussion

TGCT is a tumor that surgeons or orthopedics rarely diagnose. The international literature cites less than 10 cases of TGCT in the ankle. An important characteristic of the tumor is its slow growth, which leads to its usual diagnosis only by coincidence. Differential diagnosis has to take a number of other tumors into account, including lipoma, ganglia or fibromas. Prior to an operation, it is usually very difficult to distinguish whether the tumor is benign or malignant.

In the first case discussed in this report, the patient was suffering from pain caused by an impingement syndrome at the ventral part of his ankle. The resection of the TGCT left the patient with no discomfort or pain.

In the second case, recurrent distortions led to an advanced arthrosis in the patient's upper ankle. The patient continued to feel discomfort even after the tumor had been removed; hence, the tumor was unlikely to have caused the symptoms she experienced. Clearly, the tumor in this patient was found only by coincidence. Ligament augmentation was not performed because of advanced arthrosis in the patient's upper ankle.

The therapy of choice consists of a resection of the tumor that follows the basic principles of oncology since the tumor has to be regarded as malignant until proven otherwise [6,10-13]. A neoadjuvant or adjuvant therapy is not usually necessary [6].

The etiology of TGCT has been discussed rather controversially in the literature. Our patients presented with persisting pain in the joint after they experienced certain traumas. The tumors were only detected accidentally. In both cases, however, it remains unclear whether distortion or chronic irritation of the upper ankle may have caused or influenced the development of TGCT.

## Conclusions

Since they are rather rare, soft tissue tumors are often either taken lightly or misdiagnosed all together [3,13]. It is thus important to consider the presence of this type of tumor once common conditions such as trauma and degeneration have already been excluded.

In addition, regular follow-ups are vital due to the high recurrence rate of the tumor in up to 50% of documented cases. MRI is a very suitable technology for diagnosing and identifying a tumor. In the literature, however, no advice is given as to when the follow-up should take place. The cases discussed above were periodically reanalyzed clinically and with the use of sonography at three, six, nine and 12 months. From then on the patients were advised to attend an annual follow-up for five years afterwards. Another MRI will be done after two and five years. If the sonographic analysis shows an indication for a recurrence or if it shows unclear diagnostic findings, an MRI examination should also be performed.

## Abbreviations

TGCT: tenosynovial giant cell tumor; MRI: magnetic resonance imaging; CT: computed tomography.

## Consent

Written informed consent was obtained from the patients for publication of this case report and any accompanying images. A copy of the written consent is available for review by the Editor-in-Chief of this journal.

## Competing interests

The authors declare that they have no competing interests.

## Authors' contributions

CI and MS analyzed and interpreted the patients' examination data. HOK performed the histological examination of the specimens from the patients. LP performed the radiological examination of the patients. HRK and MS were major contributors in writing the manuscript. All authors read and approved the final manuscript.
